# Recurrent auricular haematoma following nocturnal headphone compression: a case report

**DOI:** 10.1093/jscr/rjag502

**Published:** 2026-06-25

**Authors:** Mayar Alsaqr, Enar Alotaibi, Mohammed Alkarzae

**Affiliations:** Department of Otorhinolaryngology Head and Neck Surgery, College of Medicine, King Saud University Medical City, Riyadh, Saudi Arabia; Princess Nourah Bint Abdulrahman University, Riyadh, Saudi Arabia; Otorhinolaryngology – Head and Neck Surgery, Security Forces Hospital, Riyadh, Saudi Arabia

**Keywords:** auricular hematoma, non-traumatic, headphones, case report, external ear deformity

## Abstract

Auricular hematoma typically follows blunt trauma and requires early evacuation and compression to prevent fibrocartilage deformity. Non-traumatic etiologies are uncommon. We report a 33-year-old male smoker who presented with a 1-week history of progressive left auricular swelling after sleeping overnight while wearing headphones, without any history of direct or repetitive trauma. Incision and drainage with bolster compression dressing were performed, evacuating serosanguinous fluid with minimal purulent component. Early recurrence occurred on Days 2 and 6 after the initial procedure, requiring repeat evacuation and reinforced compression dressings on both occasions. The recurrence was likely related to persistent dead space and delayed presentation. By Day 10, only minimal residual deformity remained, which remained stable at 1-year follow-up without progression. This case highlights prolonged nocturnal headphone compression as a possible non-traumatic mechanism for auricular hematoma and emphasizes the importance of close follow-up because early recurrence may occur despite appropriate management.

## Introduction

Auricular hematoma is most associated with blunt trauma, particularly in contact sports such as boxing and wrestling, in which shearing forces cause separation of the perichondrium from the underlying auricular cartilage [1, 2]. This mechanical separation disrupts the vascular supply to the avascular cartilage, resulting in haematoma formation, cartilage ischemia, and, if untreated, subsequent necrosis [3]. Delayed or inadequate management may lead to complications, including perichondritis, cellulitis, abscess formation, and the development of permanent fibrocartilaginous deformity known as cauliflower ear [1, 2]. The anterior aspect of the pinna is more frequently affected because it lacks the thicker subcutaneous tissue and muscular support present on the posterior surface, rendering the cartilage more vulnerable to shearing injury and hematoma formation [3].

Although trauma remains the predominant etiology, non-traumatic auricular hematoma is rare and has been described in association with prolonged external compression, repetitive minor mechanical irritation, and certain systemic conditions, including relapsing polychondritis and psoriasis, which have been associated with an increased susceptibility to auricular hematoma formation. In addition, spontaneous auricular haematomas have been reported in the literature [4, 5]. Anticoagulant therapy may further predispose patients to spontaneous hematoma formation or excessive blood accumulation even after minor trauma [3]. These mechanisms are thought to produce chronic low-grade shearing stress between the perichondrium and cartilage, resulting in microvascular compromise, impaired lymphatic drainage, and subsequent subperichondrial fluid accumulation similar to that observed following acute traumatic injury [5].

The principal objectives of treatment are early diagnosis, prompt evacuation of the hematoma, and prevention of reaccumulation to preserve the normal contour of the auricle [1]. Numerous therapeutic approaches have been described, including needle aspiration, incision and drainage, bolster dressings, through-and-through mattress sutures, silicone splints, buttons, and tie-down dressings [1, 2, 6]. Nevertheless, recurrence remains a significant clinical challenge, primarily due to reaccumulation within the subperichondrial space. Identified risk factors for recurrence include delayed presentation, inadequate compression, incomplete drainage, and persistent dead space following evacuation. Dalal *et al.* demonstrated lower recurrence rates in patients managed with bolster dressings following drainage procedures [7]. In addition, antibiotics with antipseudomonal coverage are frequently administered to reduce the risk of secondary perichondritis [1].

In this report, we describe a rare case of non-traumatic auricular hematoma in a 33-year-old male smoker that developed following prolonged overnight headphone use in the absence of direct trauma.

## Case presentation

A 33-year-old male smoker presented to the emergency department with a one-week history of progressive swelling of the left auricle. The patient reported that the swelling developed after sleeping while wearing headphones. He was medically free and not taking any medications. The patient denied any history of direct or minor trauma, fever, ear discharge, prior otological surgery, or insect bites. No occupational or lifestyle risk factors were identified.

**Figure 1:**
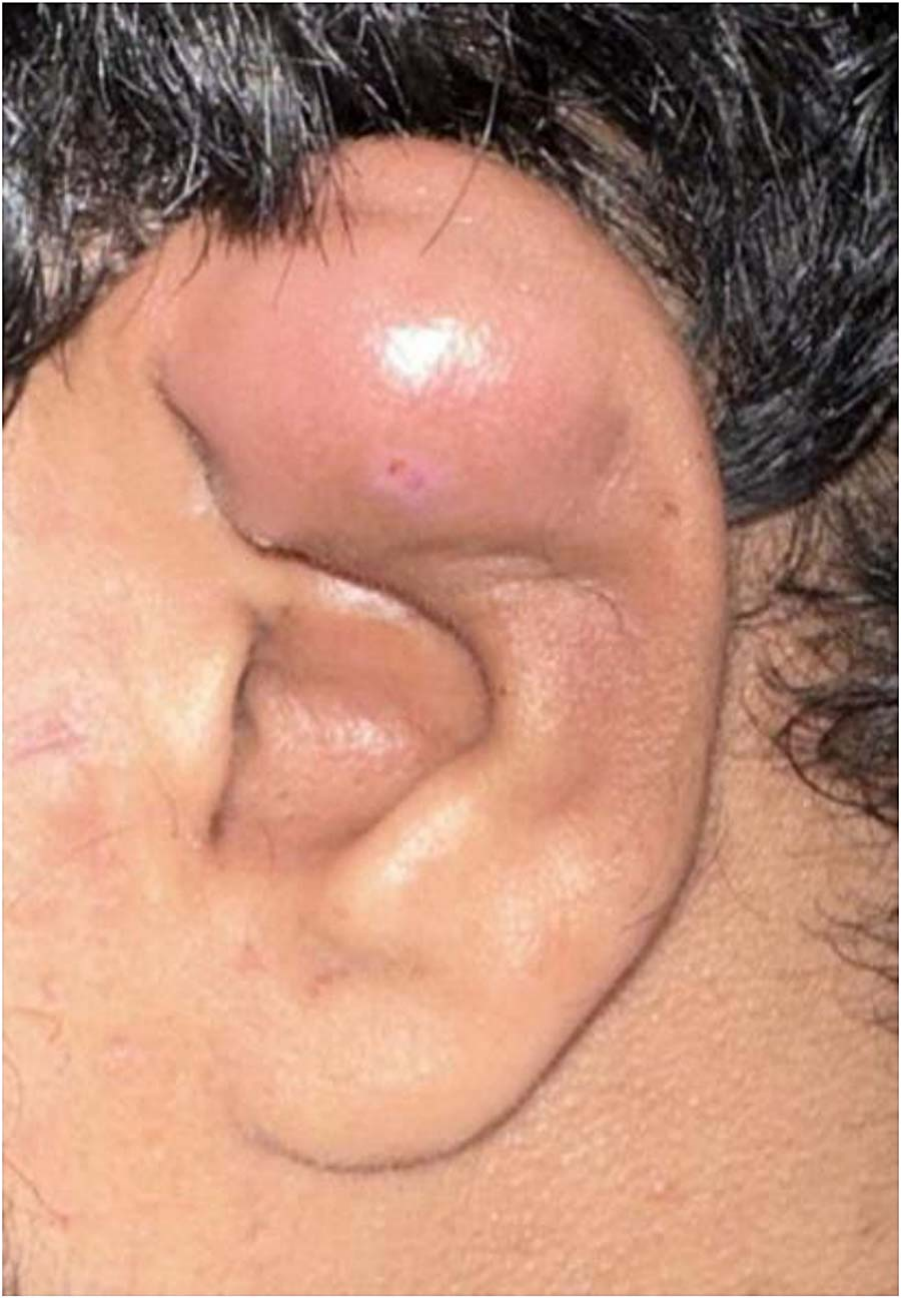
Initial presentation demonstrating a fluctuant swelling involving the anterior aspect of the auricle, consistent with auricular hematoma, with associated erythema and distortion of the normal auricular contour.

On clinical examination, a localized, fluctuant swelling measuring ~2 × 2 cm was noted over the left auricle (Fig. 1). The overlying skin was intact, with no evidence of erythema or discharge. No other palpable swellings were noted, and no regional lymphadenopathy was identified. The remainder of the physical examination was unremarkable. Laboratory investigations demonstrated a mildly elevated C-reactive protein level of 7.1 mg/L, with no evidence of leukocytosis.

A diagnosis of acute left auricular hematoma was made based on clinical history and examination findings.

Incision and drainage were performed, evacuating ~2 cc of serosanguinous fluid with minimal pus. Culture results were negative. The cavity was irrigated, Bactigras was applied, and dental roll bolster packing was secured with Prolene sutures. Empirical antibiotic therapy with oral ciprofloxacin was initiated for 2 weeks before culture results to provide coverage against *Pseudomonas aeruginosa*. The infectious diseases team was consulted, and their recommendations were incorporated into the management plan.

Reaccumulation occurred on Day 2 and Day 6, and repeat incision, drainage, and compression dressing were performed each time.

**Figure 2:**
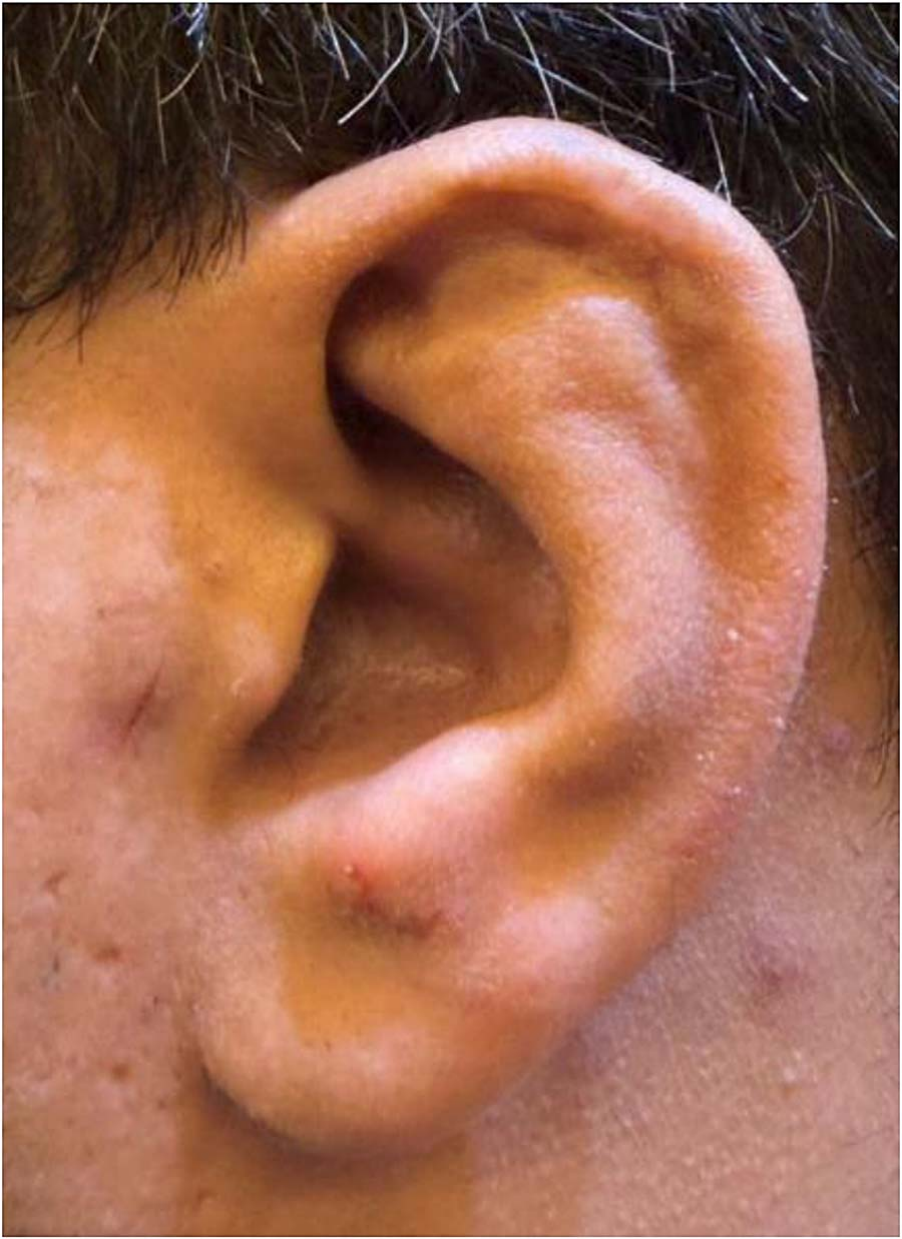
Clinical appearance on Day 10 after repeat drainage and compression dressing, demonstrating resolution of the hematoma with minimal residual deformity involving the helix and antihelix.

By Day 10, the patient demonstrated minimal residual deformity (Fig. 2), which remained stable at one-year review. The patient was lost to follow-up after the initial post-procedural period and was represented only at the one-year mark. At that time, no further progression of deformity was noted.

## Discussion

When auricular hematomas are left untreated, disruption of the vascular supply leads to cartilage ischemia and fibrocartilage proliferation, eventually resulting in permanent deformity known as cauliflower ear. Therefore, immediate intervention is required to preserve cartilage viability and prevent deformity [1, 2]. Traditional incision and drainage may still carry a risk of recurrence and deformity [8, 9]. On the other hand, techniques such as quilting sutures or alternative evacuation strategies have been described to minimize recurrence and improve outcomes [5, 6].

In our case, reaccumulation occurred on Days 2 and 6 following the initial drainage procedure. As highlighted by Al Shahrani *et al.*, recurrence may occur when residual fluid persists within multi-chambered hematoma spaces or when compression is insufficient to achieve adequate readhesion of the perichondrium to the underlying cartilage [10]. In our patient, recurrence was most likely related to persistent dead space and suboptimal compression following the initial intervention. Additionally, the ~1-week delay may have contributed to difficulty achieving complete evacuation during the first procedure. Standard incision and drainage with bolster dressing were performed on both occasions, and no further recurrence was observed after the second intervention. Nevertheless, recurrence remains a well-recognized complication of auricular hematoma despite appropriate management. Clinical improvement was ultimately achieved, with only minimal residual deformity by Day 10 following repeat drainage and more effective, sustained compression.

Empirical oral ciprofloxacin therapy was initiated to provide antipseudomonal coverage because untreated auricular hematomas may predispose to complications such as perichondritis, infection, and cartilage necrosis, as described by Greywoode *et al.* [1]. Although culture results were ultimately negative and clinical evidence of infection was minimal, treatment was continued because of the recurrent nature of the haematoma, repeated interventions, and concern for secondary perichondrial infection involving the auricular cartilage. The infectious diseases team was consulted, and their recommendations were incorporated into the management plan.

Acute trauma is the most common cause of auricular hematoma, most frequently occurring in contact sports such as wrestling and boxing. A sudden mechanical impact generates shearing forces at the perichondrial cartilage interface, resulting in rupture of subperichondrial vessels, rapid accumulation of blood, and sudden onset of swelling [3].

In contrast, non-traumatic mechanisms may represent a more insidious process involving sustained external pressure, such as prolonged headphone use or direct compression of the auricle by external devices. Rather than producing a single acute insult, this type of prolonged compression may generate repetitive low-grade shear stress at the perichondrial cartilage interface. This mechanical stress could contribute to partial separation of the perichondrium from the underlying cartilage, potentially disrupting the delicate microvascular supply to the avascular auricular cartilage. Impairment of this circulation may compromise oxygen and nutrient delivery, predisposing to vascular leakage and subsequent accumulation of blood or serosanguinous fluid within the subperichondrial space [5].

Additionally, continuous compression, particularly during sleep, may exacerbate venous outflow obstruction and impair lymphatic drainage, reducing clearance of interstitial fluid and promoting hematoma persistence.

Collectively, these mechanisms support the hypothesis that prolonged non-traumatic compression may cause sufficient microvascular injury and lymphatic dysfunction to result in auricular hematoma formation. So, this case suggests that non-traumatic causes should be considered.

To our knowledge, this is the first case to link headphone use to an auricular hematoma directly. However, similar pressure-related cases associated with prolonged mobile phone use support this mechanism and suggest that prolonged use may contribute through a combination of sustained mechanical pressure and possible local heating effects [5].

In comparison, the present case likely reflects a similar pathophysiological mechanism, where prolonged headphone use exerts sustained pressure on the auricle. On the other hand, the contribution of electromagnetic-related heating in our case remains unclear.

## Conclusion

This case highlights the importance of considering non-traumatic causes of auricular hematoma, particularly prolonged nocturnal compression from headphone use. Despite appropriate management, deformity may still occur. Further studies are needed to clarify the underlying mechanisms and associated risk factors.
